# Global population structure of *Aspergillus terreus *inferred by ISSR typing reveals geographical subclustering

**DOI:** 10.1186/1471-2180-11-203

**Published:** 2011-09-16

**Authors:** Carolyn OS Neal, Aaron O Richardson, Steven F Hurst, Anna Maria Tortorano, Maria Anna Viviani, David A Stevens, S Arunmozhi Balajee

**Affiliations:** 1 Mycotic Diseases Branch, Center for Disease Control and Prevention, 1600 Clifton Rd NE, Atlanta, GA, 30329, USA; 2 Department of Plant Biology, The University of Georgia at Athens, 2502 Miller Plant Sciences, Athens, GA 30602, USA; 3 Dipartimento di Sanità Publica-Microbiologia-Virologia, Università degli Studi di Milano via Pascal 36, 20133 Milano, Italy; 4 Division of Infectious Diseases, Department of Medicine, Santa Clara Valley Medical Center, Stanford University Medical School, 751 So. Bascom Av., San Jose, CA 95128, USA

## Abstract

**Background:**

*Aspergillus terreus *causes invasive aspergillosis (IA) in immunocompromised individuals and can be the leading cause of IA in certain medical centers. We examined a large isolate collection (n = 117) for the presence of cryptic *A. terreus *species and employed a genome scanning method, Inter-Simple Sequence Repeat (ISSR) PCR to determine *A. terreus *population structure.

**Results:**

Comparative sequence analyses of the calmodulin locus revealed the presence of the recently recognized species *A. alabamensis *(n = 4) in this collection. Maximum parsimony, Neighbor joining, and Bayesian clustering of the ISSR data from the 113 sequence-confirmed *A. terreus *isolates demonstrated that one clade was composed exclusively of isolates from Europe and another clade was enriched for isolates from the US.

**Conclusions:**

This study provides evidence of a population structure linked to geographical origin in *A. terreus*.

## Background

*Aspergillus *species are believed to be cosmopolitan organisms, existing as unstructured global populations. Species belonging to this taxon, including *A. fumigatus*, *A. terreus*, *A. flavus *and others, cause invasive aspergillosis (IA) predominantly in severely immunocompromised individuals. The majority of studies with *A. fumigatus *have demonstrated no association between genotypes and geography. Several studies employing comparative sequence analysis of different loci, including protein coding, intergenic and microsatellite containing regions, arrived at the conclusion that there was no correlation between genotype and geographical origin among *A. fumigatus *isolates [1-3]. In contrast to these observations, one study demonstrated the presence of multiple, well-supported phylogenetic clusters amongst *A. fumigatus *isolates from a collection of isolates geographically dispersed across North America [4]. The locus sequenced was a single gene encoding a putative cell surface protein, Afu3g08990 (CSP), in which polymorphisms consisted of insertions and deletions within a repeat region. The authors speculated that the presence of clusters may have been undetected previously due to the reliance on data from loci lacking sufficient polymorphisms.

*Aspergillus terreus *is the second or third most common etiological agent of IA and interestingly, appears to be the most common cause of infection in some medical centers, suggesting ecological specificity for this organism [5-7]. Previous efforts to determine population structure in *A. terreus *have been hampered by the lack of reliable methods for exploiting genetic variability to distinguish or group isolates. Balajee *et al*., employing a multi-gene sequencing approach to a large global collection of isolates previously identified as *A. terreus*, showed that no evidence of endemism existed but were able to define a genotypically distinct species, *A. alabamensis *[8]. The use of multi-locus comparative sequence analysis to explore population structure in *A. terreus *supported the existence of a single globally distributed population [8]. On the other hand, multiple studies using molecular fingerprinting methods, including RAPD, demonstrated high genotypic diversity among *A. terreus *isolates [9,10], with no evidence of endemism [9,11]. Thus, even as new species are defined within groups of isolates identified as *A. terreus*, support for the idea that *A. terreus *exists as a single, genotypically diverse, global population, lacking phylogeographic structure, continues [8-10].

A recent study investigating amphotericin B (AMB) susceptibility of a worldwide *A. terreus *collection found that isolates recovered from different parts of the world had different patterns of AMB susceptibility [12]. At that time, no attempt was made to study the association between genotypic relatedness and antifungal susceptibility in this set of isolates. In the present investigation, this *A. terreus *isolate collection was genotyped employing the highly discriminatory genome-wide DNA fingerprinting method, Inter-Simple Sequence Repeat (ISSR) PCR [13] to (a) assess the use of this fingerprinting method for discriminatory genotyping of *A. terreus*; (b) evaluate the association between AMB susceptibility and genotype in this global collection of isolates; and (c) attempt to map geography onto genotypically related clusters of isolates. Results of this study revealed the possible global sub-structuring of genotypes and the presence of the recently described cryptic species *A. alabamensis *in Italy.

## Methods

### Fungal Strains and genomic DNA Isolation

A total of 117 clinical *A. terreus *isolates originating from France or Belgium (28 isolates), Italy (46 isolates), and the Eastern (22 isolates) and Western (21 isolates) United States were available for analyses from the previously performed study [12]. All isolates were subcultured on Sabouraud Dextrose Agar (SDA) plates in preparation for genomic DNA isolation.

For genomic DNA extraction, fungal material was removed from plates and disrupted using an Omni mixer (Omni International, Warrenton, VA) in the presence of ATL buffer from the DNeasy Blood and Tissue Kit (Qiagen, Valencia, CA) containing 1 mg/ml proteinase K (Sigma, St. Louis, MO). The disrupted material was incubated at 55°C for one hour with vortexing every 15 min. DNA was isolated using the DNeasy Blood and Tissue Kit (Qiagen, Valencia, CA) according to the manufacturer's protocol. Genomic DNA quality was checked with electrophoresis in a 1% agarose gel (Roche, Manheim, Germany) and quantity was measured with the nanodrop spectrophotometer at a wavelength of 260A (Thermo Fisher Scientific, Pittsburgh, PA).

### Comparative Sequence Analysis of the calmodulin gene

Portions of the calmodulin locus (*calM*) were PCR amplified and sequenced as previously described [8]. The resultant nucleotide sequences were edited with SeqMan Pro Ver 8.0.2 software (DNASTAR, Inc., Madison, WI). Edited sequences were aligned using Bioedit Sequence Alignment Editor Ver 7.0.9.0. Reference sequences were downloaded from GenBank and the software program GARLI [Genetic Algorithm for Rapid Likelihood Inference] was used to generate the maximum likelihood (ML) tree [14].

### Development of ISSR Fingerprinting Method

The ISSR primers were designed to flank di-, tri- and tetra-nucleotide repeats. A total of ten repeat primers were synthesized: two di-nuclotide [DDB(nn)_8_], five trinucleotide [DDB(nnn)_5_], and three tetranuclotide [DDB(nnnn)_4_] (capital letters denote degenerate sites: B denotes nucleotides c, g, or t; D denotes a, g, or t; subscripts indicate the number of repeats) and 5' labeled with 6-carboxyfluorescein dye (6-FAM) at the Centers for Disease Control and Prevention Biotechnology Core Facility (Atlanta, GA) (Table 1).

**Table 1:** ISSR primers designed for this study

Primer	Sequence	Repeat Type
**ISSR_7**	**DDB(agg)_5_**	**Trinucleotide**
ISSR_8	DDB(cag)_5_	Trinucleotide
**ISSR_9**	**DDB(gag)5**	**Trinucleotide**
**ISSR_10**	**DDB(ctc)_5_**	**Trinucleotide**
ISSR_11	DDB(gtg)_5_	Trinucleotide
ISSR_12	DDB(aacg)_4_	Tetranucleotide
**ISSR_13**	**DDB(cgca)_4 _**	**Tetranucleotide**
ISSR_14	DDB(gcca)_4_	Tetranucleotide
ISSR_15	DDB(ct)_8_	Dinucleotide
ISSR_16	DDB(ca)_8_	Dinucleotide

Capital letters in ISSR primer sequences denote degenerate sites: B denotes nucleotides c, g, or t; D denotes a, g, or t. Subscripts indicate the number of repeats. **Bold **lettering indicates the primers used for fingerprinting.

Initially, ten ISSR primers were tested for their ability to generate reproducible, complex fingerprinting patterns on a panel of 40 *A. terreus *isolates randomly selected from the global isolate collection. For PCR amplification, 3-5 μl of genomic DNA was used as the template in a final reaction volume of 25 μl consisting of PCR buffer (10 mM Tris-HCl, 1.5 mM MgCl_2_, 50 mM KCl, pH 8.3); 0.2 mM each of dATP, dGTP, dCTP, and dTTP; 2 pmol of a single primer; and 1.3 U of Taq DNA polymerase (Roche Applied Science, Mannheim, Germany). Amplification was performed in a GeneAmp PCR system 9700 thermocycler (Applied Biosystems, Carlsbad, CA). Initial denaturation at 95°C for 5 min was followed by 36 cycles of 95°C for 30 s, 50°C for 45 s, and 72°C for 2 min. The last cycle was followed by a final extension at 72°C for 7 min. Fluorescently labeled PCR products were separated by capillary electrophoresis on an ABI 3130 DNA analyzer (Applied Biosystems, Carlsbad, CA). Briefly, 0.5 μl of a 1:10 dilution of PCR product was added to 0.25 μl GeneScanTM 1200 LIZ internal size standard and 9.25 μl Hi-Di formamide (Applied Biosystems, Carlsbad, CA). The 10 μl samples were denatured by heating to 95°C for 3 min., cooled and run on a 50 cm array in the POP-7 polymer matrix using the 1200LIZ run module.

Four of the ten primers tested produced complex, reproducible, banding patterns over multiple PCR reactions and a series of DNA concentrations, and these four ISSR primers were therefore selected for the analysis of the remaining sequence-confirmed *A. terreus *isolates.

### Subtyping of the global *A. terreus *isolates

Fingerprints for all of the sequence-confirmed *A. terreus *isolates were generated using four ISSR primers that were selected after initial screening as described above. GeneMapper v4.0 (Applied Biosystems, Carlsbad, CA) was used to assign fragment sizes to the PCR products. Fragments identified using GeneMapper software were converted to binary data with a "0" representing the absence and a "1" representing the presence of an allele. The binary strings of data representing the fingerprint generated by each primer were concatenated in Excel (Microsoft Corporation, Redmond, WA) to form a single, continuous, binary string incorporating the results from all primers. Alleles that appeared in all or fewer than 10% of isolates were excluded from the analysis. Phylogenetic trees and Bayesian clusters were generated from identical binary data sets.

### Phylogenetic Analysis of ISSR data

Neighbor-joining (NJ) trees were generated by PAUP [Phylogenetic Analysis Using Parsimony (and Other Methods)] [15]. PHYLIP [Phylogeny Inference Package] [16] was used to produce the parsimony tree. Bayesian clustering was performed using the program STRUCTURE [17].

## Results

### Species Confirmation

**Figure 1:**
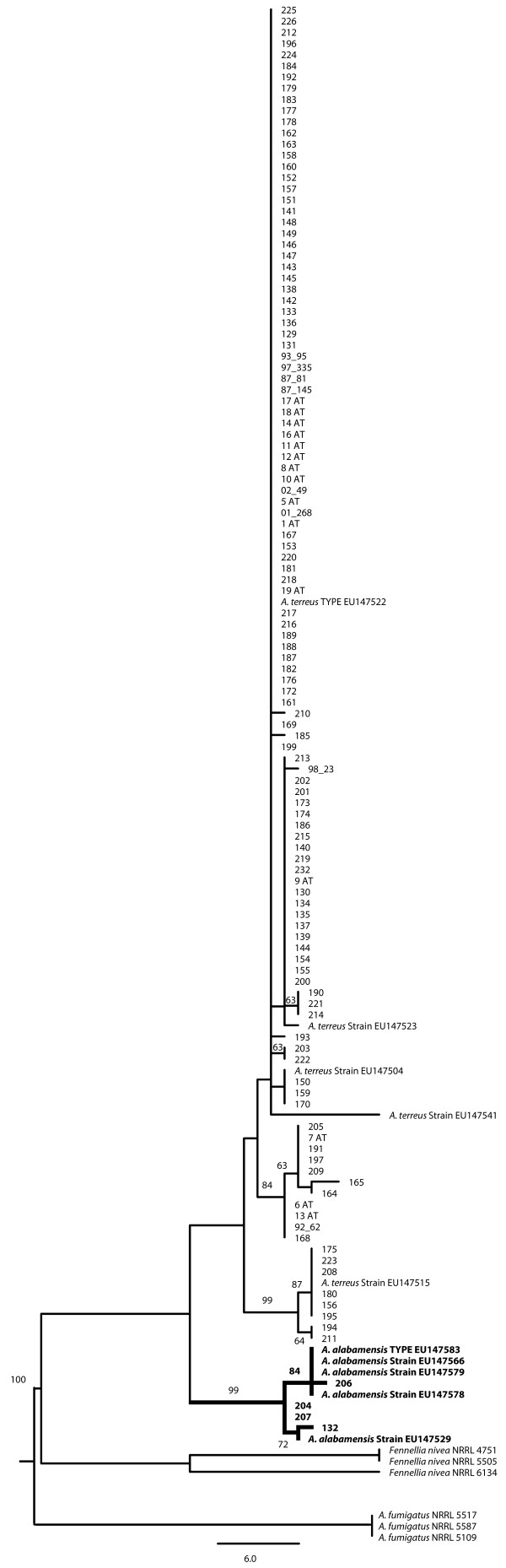
**Maximum Liklihood Tree from Calmodulin Sequence of Aspergillus species**. Maximum likelihood tree of partial nucleotide sequences of calmodulin gene region obtained for all isolates and reference *A. terreus *and *A. alabamensis *sequences from GenBank. *A. alabamensis *isolates and reference sequences are in bold. Bootstrap values above 50% from 1000 iterations are noted on nodes.

The ML tree was generated using 484 contiguous bases of aligned sequence from the *calM *locus of the 117 *A. terreus *isolates and additional reference section *Terrei *sequences acquired from GenBank. One hundred and thirteen isolates clustered with the reference *A. terreus *isolates and four isolates, three from the Eastern United States and one from Italy, grouped with the *A. alabamensis *type isolate (Figure 1).

### ISSR Fingerprinting of the Global *A. terreus *Isolates

On testing ten ISSR primers using a subset of forty *A. terreus *isolates, it was found that four primers were suitable for generating robust fingerprints for *A. terreus*: three trinucleotide repeat flanking primers and a single tetranuclotide repeat flanking primer (ISSR 7, 9, 10 and 13 respectively) (Table 1). These four ISSR primers were used to generate fingerprints for all of the sequence-confirmed *A. terreus *isolates. The *A. alabamensis *isolates were not fingerprinted. ISSR subtyping of 113 *A. terreus *revealed 111 unique genotypes with only two isolates, both from the same center in the Eastern United States, demonstrating identical fingerprinting patterns.

**Figure 2:**
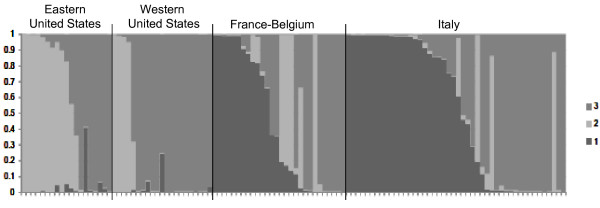
**STRUCTURE grouping of *A. terreus *isolates**. Inferred population structure of *A. terreus *isolates from STRUCTURE analysis of ISSR data. Each isolate is represented by a vertical bar partitioned into shaded segments representing the isolate's estimated proportion of ancestry from each of three clusters defined by STRUCTURE.

**Figure 3:**
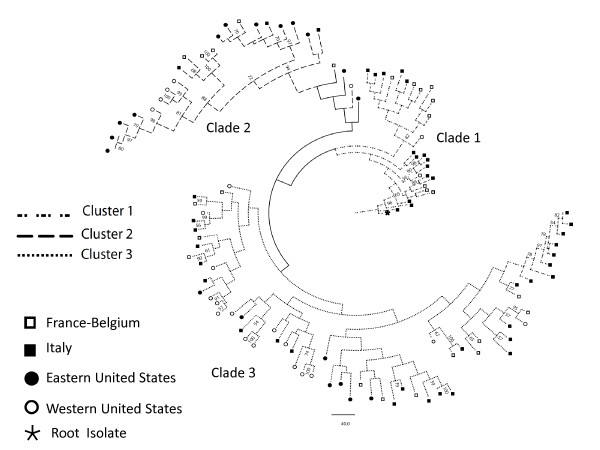
**Neighbor joining tree from ISSR fingerprints of *A. terreus *isolates**. Phylogenetic relationship among *A. terreus *isolates inferred by ISSR fingerprints using the Neighbor joining algorithm. The tree is rooted with the outgroup *Aspergillus fumigatus*. Bootstrap values above 50% from 1000 iterations are noted on nodes. Lines indicate isolate affiliation with clusters defined by STRUCTURE. Filled and open circles and squares indicate geographic origin of isolates.

Data from the ISSR fingerprints were analyzed using three phylogenetic algorithms. For the purposes of this manuscript, clusters were defined as groupings generated using Bayesian posterior probability while clades were defined as groupings generated using NJ and parsimony. Bayesian clustering of the ISSR data using STRUCTURE supported the presence of three clusters among the isolates (Figure 2). Both Maximum parsimony (not shown) and NJ trees (Figure 3) were in agreement with the clusters defined by STRUCTURE. Although there was no significant bootstrap support for two of the clades on the NJ tree [1] and [3], support for clade 2 was 94%. Clade 1, composed exclusively of isolates from Europe, contained 27 of the 113 isolates. Sixteen isolates in this European clade were from Italy and 11 isolates were from Belgium or France. The type of line in Figure 3 indicates the cluster membership of each isolate on the NJ tree and illustrates the correlation between clades and clusters. Bayesian clustering of the ISSR data also supported the existence of the European clade. (Figure 3) The European cluster 1, as revealed by STRUCTURE, contained 34 isolates while the European clade 1 (NJ and MP algorithms) contained 27 of the same isolates. Many European isolates did not, however, fall into the exclusively European cluster or clade.

A significant relationship existed between geography and cluster membership (X^2 ^= 48.2, d.f. = 6, p < 0.001), driven primarily by cluster 1 being composed only of isolates from Europe, as well as cluster 2 accounting for the majority [12 of 19] of the sequence-confirmed Eastern U.S. *A. terreus *isolates (Figure 2). The patterns of cluster membership in the two US populations were similar to each other and quite different from the pattern shared by the two European populations (Figure 2). There were nine isolates in which the majority contribution from any cluster was less than 0.66, suggesting that these isolates did not consistently fall into any one cluster. These isolates were excluded from any single cluster due to their high levels of inferred admixture.

### Susceptibility testing to AMB

Susceptibility testing to AMB for all the isolates analyzed in this investigation was available through a previous study summarized in Table 1 of Tortorano et al [12]. The isolates in each of the three clusters varied in mean MIC values (0.78, 1.29 and 0.86 mg/L for clusters 1, 2 and 3 respectively (Table 2). The MIC values of cluster 1 and 2 were significantly different (2-tailed t-test, p < 0.05,), but the difference between clusters 1 and 3, and 2 and 3 were not statistically significant. The isolates from different geographic locations also varied in mean MIC values but were not significantly different (data not shown).

**Table 2:** Mean MIC for Structure Defined Clusters

CLUSTER (→) Geographic Origin (↓)	1	2	3	Total isolates
**Italy**	22 (1)	3	17 (2)	45
**France-Belgium**	11	4 (1)	10 (2)	28
**Eastern US**	0	10 (2)	6 (1)	19
**Western US**	0	5	16	21
**MEAN MIC (AMB) mg/L (→)**	0.78	1.29	0.86	113

Mean MIC for STRUCTURE defined clusters of sequence-confirmed *A. terreus *isolates. Numbers in parenthesis denote isolates in which the majority contribution from any cluster was less than 0.66.

## Discussion

Extensive genotypic diversity has long been known in *A. terreus*, and recently a cryptic species, *A alabamensis*, was discovered among isolates originally identified as *A. terreus *[8]. In the current study, we report the presence of four *A. alabamensis *isolates, identified by comparative sequence analysis of a previously characterized single locus gene (*calM*), from a collection of clinical isolates defined as *A. terreus*. Three *A. alabamensis *isolates were recovered from North America and one originated from Italy, making this the first reported *A. alabamensis *isolate recovered outside of North America. In contrast to a previous study that found that *A. alabamensis *had decreased in vitro susceptibility to AMB [8], all four *A. alabamensis *isolates recovered in this study had similar MIC patterns against AMB as compared to *A. terreus *(data not shown). None of the *A. alabamensis *isolates recovered in this study were colonizers (David Stevens, personal communication), a finding that was different from the study of Balajee et al. [8].

It has been postulated that unique *A. terreus *genotypes may occupy particular environmental niches associated with certain geographical areas. To test this hypothesis, Lass-Florl et al. [9] conducted a molecular epidemiological study using RAPD which explored the genotypes of clinical isolates recovered from two medical centers that more frequently reported *A. terreus *infections. Results of this study reported a great diversity of genotypes among isolates from both centers and revealed no evidence of endemicity among the isolates at either center. Another study investigating in vitro activity of AMB against a large global collection of clinical isolates suggested that isolates from different parts of the world could have differences in AMB susceptibility [12]. Tortorano et al. [12] found that of the four geographic locations where isolates originated, the average MIC of the isolates from the Eastern United States were statistically different from those of the isolates from the other three geographical regions namely, Italy, France-Belgium, and the Western United States, suggesting a possible association between geography and MIC. We postulated that an association between geography and genotype may also exist in this population and that ISSR fingerprinting may be a superior way to assess population structure in these isolates.

Results of our study demonstrated high genotypic diversity within these isolates with only two isolates displaying identical fingerprinting patterns. In spite of this high genotypic diversity, sufficient common markers existed between isolates to group them into distinct clades supported by multiple phylogenetic methods. Specifically, Bayesian clustering in the program STRUCTURE revealed 3 distinct clusters of isolates that were in agreement with the clades inferred by NJ. Cluster 2 (Figure 2 and 3) generated by STRUCTURE shares the isolates in clade 2 of the NJ tree which had the highest bootstrap support of any clade. This suggests that these isolates share alleles that are less enriched in isolates from the other two clades, and thus may be the most ancient group. Isolates in cluster 1 were restricted to Europe, while isolates in cluster 2 were most commonly recovered from the U.S., and cluster 3 included isolates recovered globally. There were nine isolates with high levels of inferred admixture that did not belong to any single cluster. It is tempting to speculate that human activities may have facilitated the global distribution of cluster 3 and the admixture between populations.

Clustering of isolates from the same sampling area suggests a link between genetic similarity and geographic origin in a population of organisms previously believed to lack endemism. Additional isolates from both clinical and environmental sources obtained from diverse geographical regions will need to be rigorously examined to verify the endemism suggested by our study. An expanded population structure analysis including isolates with more complete epidemiological data could lend predictive power about antifungal susceptibility to future studies. In contrast to the above finding, the relationship between population structure and AMB susceptibility was small. This could be attributable to the sample size being too small or to the lack of an association between in vitro antifungal susceptibilities and geographical origin.

## Conclusions

Multiple studies have demonstrated that *A. terreus *is the predominant etiological agent of IA in certain medical centers around the world including those in Houston, Texas, and Innsbruck, Austria [5,9,18]. Molecular examination of isolates from these centers showed no endemism and the authors concluded that other factors including levels of immunosuppression and previous antifungal use in the host, could, in part, be responsible for the prevalence of *A. terreus *in these medical centers. We have demonstrated in this study, using a discriminatory molecular method, a different set of globally derived isolates and rigorous phylogenetic analysis of the resulting data, that *A. terreus *may exhibit endemism. It remains to be seen if isolates recovered from other parts of the world, importantly from centers in Texas and Austria, cluster according to geographical location. Such molecular characterization of medically important fungi could be pivotal in understanding the ecology, acquisition and transmission of these organisms.

## Disclaimer

The findings and conclusions in this article are those of the author(s) and do not necessarily represent the views of the CDC.

## Authors' contributions

COSN performed DNA fingerprinting, participated in the phylogenetic analyses and manuscript drafting. AOR performed statistical and participated in the phylogenetic analysis. SFH participated in DNA fingerprinting and sequence alignment. AMT and MAV provided isolates used in the study and contributed to the draft manuscript. DAS coordinated the study and contributed to the draft manuscript. SAB designed and supervised the study and wrote the final manuscript. All authors read and approved this manuscript.
